# Deep Learning-Based Concurrent Brain Registration and Tumor Segmentation

**DOI:** 10.3389/fncom.2020.00017

**Published:** 2020-03-20

**Authors:** Théo Estienne, Marvin Lerousseau, Maria Vakalopoulou, Emilie Alvarez Andres, Enzo Battistella, Alexandre Carré, Siddhartha Chandra, Stergios Christodoulidis, Mihir Sahasrabudhe, Roger Sun, Charlotte Robert, Hugues Talbot, Nikos Paragios, Eric Deutsch

**Affiliations:** ^1^Gustave Roussy-CentraleSupélec-TheraPanacea Center of Artificial Intelligence in Radiation Therapy and Oncology, Gustave Roussy Cancer Campus, Villejuif, France; ^2^Université Paris-Saclay, Institut Gustave Roussy, Inserm, Molecular Radiotherapy and Innovative Therapeutics, Villejuif, France; ^3^Gustave Roussy Cancer Campus, Department of Radiation Oncology, Villejuif, France; ^4^Université Paris-Saclay, CentraleSupélec, Mathématiques et Informatique pour la Complexité et les Systèmes, Gif-sur-Yvette, France; ^5^Université Paris-Saclay, CentraleSupélec, Inria, Centre de Vision Numérique, Gif-sur-Yvette, France; ^6^Université Paris-Saclay, Institut Gustave Roussy, Inserm, Predictive Biomarkers and Novel Therapeutic Strategies in Oncology, Villejuif, France

**Keywords:** brain tumor segmentation, deformable registration, multi-task networks, deep learning, convolutional neural networks

## Abstract

Image registration and segmentation are the two most studied problems in medical image analysis. Deep learning algorithms have recently gained a lot of attention due to their success and state-of-the-art results in variety of problems and communities. In this paper, we propose a novel, efficient, and multi-task algorithm that addresses the problems of image registration and brain tumor segmentation jointly. Our method exploits the dependencies between these tasks through a natural coupling of their interdependencies during inference. In particular, the similarity constraints are relaxed within the tumor regions using an efficient and relatively simple formulation. We evaluated the performance of our formulation both quantitatively and qualitatively for registration and segmentation problems on two publicly available datasets (BraTS 2018 and OASIS 3), reporting competitive results with other recent state-of-the-art methods. Moreover, our proposed framework reports significant amelioration (*p* < 0.005) for the registration performance inside the tumor locations, providing a generic method that does not need any predefined conditions (e.g., absence of abnormalities) about the volumes to be registered. Our implementation is publicly available online at https://github.com/TheoEst/joint_registration_tumor_segmentation.

## 1. Introduction

Brain tumors and more specifically gliomas as one of the most frequent types, are across the most dangerous and rapidly growing types of cancer (Holland, 2002). In clinical practice, multi-modal magnetic resonance imaging (MRI) is the primary method of screening and diagnosis of gliomas. While gliomas are commonly stratified into Low grade and High grade due to different histology and imaging aspects, prognosis and treatment strategy, radiotherapy is one of the mainstays of treatment (Stupp et al., 2014; Sepúlveda-Sánchez et al., 2018). However, radiotherapy treatment planning relies on tumor manual segmentation by physicians, making the process tedious, time-consuming, and sensitive to bias due to low inter-observer agreement (Wee et al., 2015).

In order to overcome these limitations, numerous methods have been proposed recently that try to provide tools and algorithms that will make the process of gliomas segmentation automatic and accurate (Parisot et al., 2016; Zhao et al., 2018). Toward this direction, the multimodal brain tumor segmentation challenge (BraTS) (Menze et al., 2015; Bakas et al., 2017a,b,c) is annually organized, in order to highlight efficient approaches and indicate the way toward this challenging problem. In recent years, most of the approaches that exploit BraTS have been based on deep learning architectures using 3D convolutional neural networks (CNNs) similar to VNet (Milletari et al., 2016). In particular, the best performing approaches use ensembles of deep learning architectures (Kamnitsas et al., 2018; Zhou et al., 2018), with autoencoder regularization (Myronenko, 2018) or they even combine deep learning architectures together with algorithms, such as conditional random fields (CRFs) (Chandra et al., 2019). Other top-performing methods in the BraTS 2017 and 2018 challenges used cascaded networks, multi-view and multi-scale approaches (Wang et al., 2017), generic UNet architecture with data augmentation and post-processing (Isensee et al., 2018), dilated convolutions and label uncertainty loss (McKinley et al., 2018), and context aggregation and localization pathways (Isensee et al., 2017). A more detailed comparison and presentation of competing methods in recent BraTS challenges is presented and summarized in Bakas et al. (2018).

Image registration is a challenging task for medical image analysis in general and for rapidly evolving brain tumors in particular, where longitudinal assessment is critical. Image registration seeks to determine a transformation that will map two volumes (source and reference) to the same coordinate system. In practice, we seek a volume mapping function that changes the coordinate system of the source volume into the coordinate system of the reference volume. Among the different types of methods employed in medical applications, deformable or elastic registration is the most commonly used (Sotiras et al., 2013). Linear methods are an alternative but in that case a linear global transformation is sought for the entire volume. Deformable registration has been addressed with a variety of methods, including for example surface matching (Postelnicu et al., 2009; Robinson et al., 2018) or graph based approaches (Glocker et al., 2009). These methods have been extended to address co-registration of multiple volumes (Ou et al., 2011). Moreover, some of the most popular methods traditionally used for the accurate deformable registration include (Avants et al., 2008; Klein et al., 2009; Shi et al., 2013). Recently a variety of deep learning based methods have been proposed, reducing significantly the computational time but maintaining the accuracy and robustness of the registration (Christodoulidis et al., 2018; Dalca et al., 2018). In particular, the authors in Dalca et al. (2018) presented a deep learning framework trained for atlas-based registration of brain MR images, while in Christodoulidis et al. (2018) the authors present a scheme for a concurrent linear and deformable registration of lung MR images. However, when it comes to anatomies that contain abnormalities, such as tumoral areas, these methods fail to register the volumes at certain locations, due to lack of similarity between them. This often leads to distortions in and around the tumor regions in the deformed image.

To overcome this problem, in this paper, we propose a dual deep learning based architecture that addresses registration and tumor segmentation simultaneously, relaxing the registration constraints inside the predicted tumor areas, providing displacements and segmentation maps at the same time. Our framework bears concept similarities with the work presented in Parisot et al. (2012) where a Markov Random Field (MRF) framework has been proposed to address both of tumor segmentation and image registration jointly. Their method required ~6 min for the registration of one pair and the segmentation of one class tumor region was performed with handcrafted features and classical machine learning techniques using only one MRI modality. Moreover, there are methods in the literature that try to address the problem of registration of brain tumor MRI by registering on atlases or MRIs without tumoral regions (Gooya et al., 2010, 2012). Here, we introduce a highly scalable, modular, generic, and precise 3D-CNN for both registration and segmentation tasks and provide a computationally efficient and accurate method for registering any arbitrary subject involving possible abnormalities. To the best of our knowledge this is the first time that a joint deep learning-based architecture is presented, showing very promising results in two publicly available datasets for brain MRI. The proposed framework provides a very powerful formulation by introducing the means to elucidate clinical or functional trends in the anatomy or physiology of the brain via the registration branch. It further enables the modeling and the detection of brain tumor areas due to the synergy with the segmentation branch.

## 2. Materials and Methods

**Figure 1:**
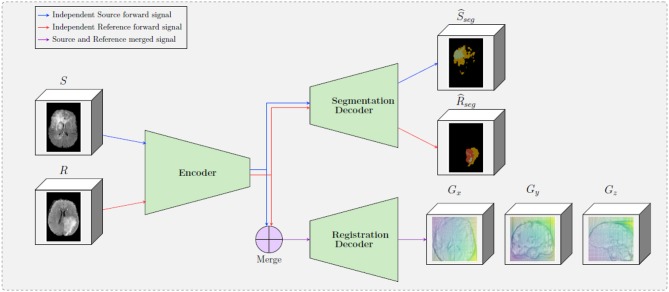
A schematic representation of the proposed framework. The framework is composed by two decoders, one which provides tumor segmentation masks for both *S* and *R* images, and one the provides the optimal displacement grid *G* that will accurately map the *S* to the *R* image. The merge bloc will combine the forward signal of the source input and the reference input (which are forwarded independently in the encoder).

Consider a pair of medical volumes from two different patients—a source *S*, and a reference *R* together with their annotations for the tumor areas (*S*_*seg*_ and *R*_*seg*_). The framework consists of a bi-cephalic structure with shared parameters, depicted in Figure 1. During training the network uses as input a source *S* and a reference *R* volumes and outputs their brain tumor segmentation masks ${\hat{S}}_{seg}$ and ${\hat{R}}_{seg}$ and the optimal elastic transformation *G* which will project or map the source volume to the reference volume. The goal of the registration part is to find the optimal transformation to transform the source *S* to the reference *R* volume. In this section, we present the details for each of the blocks as well as our final formulation for the optimization.

### 2.1. Shared Encoder

One of the main differences of the proposed formulation with other registration approaches in the literature is the way that the source and reference volumes are combined. In particular, instead of concatenating the two initial volumes, these volumes are independently forwarded in a unique encoder, yielding two sets of features maps (called *latent codes*) *C*_source_ and *C*_reference_ for the source and the reference volumes, respectively. These two codes are then independently forwarded into the segmentation decoder, providing the predicted segmentation maps ${\hat{S}}_{seg}$ and ${\hat{R}}_{seg}$. Simultaneously, the two codes are merged before being forwarded in the registration decoder—this operation is depicted in the “Merge” block in Figure 1. The motivation behind adopting this strategy is based on forcing the encoder to extract meaningful representations from individual volumes instead of a pair of volumes. This is equivalent to asking the encoder discovering a template, “deformation-free” space for all volumes, and encoding each volume against this space (Shu et al., 2018), instead of decoding the deformation grid between every possible pair of volumes. Besides, from the segmentation point of view, there are no relationship between the tumor maps of the source volume and the reference volume, so the codes to be forwarded into the segmentation decoder should not depend on each other.

We tested two merging operators, namely concatenation and subtraction. Both source and reference images are 4*D* volumes whose first dimension corresponds to the 4 different MRI modalities that are used per subject. After the forward to the encoder, the codes *C*_source_ and *C*_reference_ are also 4D volumes with the first dimension corresponding to *n*_*f*_, which is the number of convolutional filters of the last block of the encoder. Before *C*_source_ and *C*_reference_ are inserted into the registration decoder, they are merged, outputting one 4D volume of size 2 × *n*_*f*_ in the case of the concatenation, and of size *n*_*f*_ for the elementwise subtraction operator, both leaving the rest of the dimensions unchanged. In particular, the subtraction presents the following natural properties for every coding image *C*_*I*_:

- $\forall C_{I}\in {\mathbb{R}}^{n}:Merge\left(C_{I},C_{I}\right)=0$
- $\forall C_{I},C_{J}\in {\mathbb{R}}^{n}\times {\mathbb{R}}^{n}:Merge\left(C_{I},C_{J}\right)=-Merge\left(C_{J},C_{I}\right)$

### 2.2. Brain Tumor Segmentation Decoder

Inspired by the latest advances reported on the BraTS 2018 dataset, we adopt a powerful autoencoder architecture. The segmentation and registration decoders share the same encoder (section 2.1) for feature extraction and they provide brain tumor segmentation masks (${\hat{S}}_{seg}$ and ${\hat{R}}_{seg}$) for the source and the reference images. These masks refer to valuable information about the regions that cannot be registered properly as there is no corresponding anatomical information on the pair. This information is integrated into the optimization of the registration component, relaxing the similarity constraints and preserving to a certain extent the geometric properties of the tumor.

Variety of loss functions have been proposed in the literature for the semantic segmentation of 3D medical volumes. In this paper, we performed all our experiments using weighted categorical cross-entropy loss and optimizing three different segmentation classes for the tumor area as provided by the BraTS dataset. In particular,

(1) $$\begin{array}{r} L_{seg}=CE\left(S_{seg},{\hat{S}}_{seg}\right)+CE\left(R_{seg},{\hat{R}}_{seg}\right) \end{array}$$

where *CE* denotes the weighted cross entropy loss. The cross entropy is calculated for both the source and reference images and the overall segmentation loss is the sum of the two. Here we should note that different segmentation losses can be applicable as for example the dice coefficient (Sudre et al., 2017), focal loss (Lin et al., 2017), etc.

### 2.3. Elastic Registration Decoder

In this paper, the registration strategy is based on the one presented in Christodoulidis et al. (2018), with the main component being the 3D spatial transformer. A spatial transformer deforms (or warps) a given image *S* with a deformation grid *G*. It can be represented by the operation,

$$\begin{array}{l} D=W\left(S,G\right), \end{array}$$

where W(·,G) indicates a sampling operation W under the deformation *G* and *D* the deformed image. The deformation is hence fed to the transformer layer as sampling coordinates for a backward trilinear interpolation sampling, adapting a strategy similar to Shu et al. (2018). The sampling process is then described by

$$\begin{array}{l} D\left(\vec{p}\right)=W\left(S,G\right)\left(\vec{p}\right)=\underset{\vec{q}}{\sum }S\left(\vec{q}\right)\underset{d}{\prod }\max\left(0,1-|{\left[G\left(\vec{p}\right)\right]}_{d}-{\vec{q}}_{d}|\right), \end{array}$$

where $\vec{p}$ and $\vec{q}$ denote pixel locations, *d* ∈ {*x, y, z*} denotes an axis, and ${\left[G\left(\vec{p}\right)\right]}_{d}$ denotes the *d*-component of $G\left(\vec{p}\right)$. Moreover, instead of regressing per-pixel displacements, we predict a matrix Ψ of spatial gradients between consecutive pixels along each axis. The actual grid *G* can then be obtained by applying an integration operation on Ψ along the *x*-, *y*-, and *z*-axes, which is approximated by the cumulative sum in the discrete case. Consequently, two pixels $\vec{p}$ and $\vec{p}+1$ will have moved closer, maintained distance, or moved apart in the warped image, if ${\text{Ψ}}_{\vec{p}}$ is respectively < 1, = 1, or > 1.

### 2.4. Network Architecture

Our network architecture is a modified version of the fully convolutional VNet (Milletari et al., 2016) for the underlying encoder and decoders parts, maintaining the depth of the model and the rest of the filter's configuration unchanged. The model, whose computational graph is displayed in Table 1, comprises several sequential residual convolutional blocks made of one to three convolutional layers, followed by downsampling convolutions for the encoder part and upsampling convolutions for the decoder part. We replaced the initial 5 × 5 × 5 convolutions filter-size by 3 × 3 × 3 in order to reduce the number of parameters without changing the depth of the model, and also replace PReLu activations by ReLU ones. In order to speed up its convergence, the model uses residual connections between each encoding and corresponding decoding stage for both the segmentation and the registration decoder. This allows every layer of the network, particularly the first ones, to be trained more efficiently since the gradient can flow easier from the last layers to the first ones with less vanishing or exploding gradient issues. The encoder part deals with 4-inputs per volume, representing the four different MRI modalities that are available on the BraTS dataset, an extra 1 × 1 × 1 convolution is added to fuse the initial modalities. Moreover, the architecture contains 2 decoders of identical blocks, 1 dedicated to the segmentation of tumors for the source and reference image and 1 dedicated to the optimal displacement that will map the source to the reference image.

**Table 1:** Layer architectures of the shared encoder, the segmentation decoder and the registration decoder.

**Name**	**Input**	**Res. input**	**Operations**	**Output shape**
**ENCODER**				
Enc^1^	4D MRI		Conv_1,8_, ReLU, (Conv_3,8_, ReLU), AddId,	(144, 208, 144, 8)
Enc^2^	Enc^1^		Conv_2,16_, ReLU, (Conv_3,16_, ReLU)*2, AddId	(72, 104, 72, 16)
Enc^3^	Enc^2^		Conv_2,32_, ReLU, (Conv_3,32_, ReLU)*3, AddId	(36, 52, 36, 32)
Enc^4^	Enc^3^		Conv_2,64_, ReLU, (Conv_3,64_, ReLU)*3, AddId	(18, 26, 18, 64)
Enc^5^	Enc^4^		Conv_2,128_, ReLU, (Conv_3,128_, ReLU)*3, AddId	(9, 13, 9, 128)
**SEGMENTATION DECODER**				
${\text{Dec}}_{seg}^{4}$	Enc^5^	Enc^4^	DeConv_2,64_,ReLU, ResConc, (Conv_3,64_, ReLU)*3, AddId	(18, 26, 18, 64)
${\text{Dec}}_{seg}^{3}$	${\text{Dec}}_{seg}^{4}$	Enc^3^	DeConv_2,32_, ReLU, ResConc, (Conv_3,32_, ReLU)*3, AddId	(36, 52, 36, 32)
${\text{Dec}}_{seg}^{2}$	${\text{Dec}}_{seg}^{3}$	Enc^2^	DeConv_2,16_, ReLU, ResConc, (Conv_3,16_, ReLU)*2, AddId	(72, 104, 72, 16)
${\text{Dec}}_{seg}^{1}$	${\text{Dec}}_{seg}^{2}$	Enc^1^	DeConv_2,8_, ReLU, ResConc, (Conv_3,8_, ReLU), AddId	(144, 208, 144, 8)
${\text{Dec}}_{seg}^{0}$	${\text{Dec}}_{seg}^{1}$		Conv_1,4_, Softmax	(144, 208, 144, 4)
**REGISTRATION DECODER**				
Merge	${\text{Enc}}_{R}^{i}$, ${\text{Enc}}_{S}^{i}$		For all $1\leq i\leq 5,{\text{MEnc}}^{i}={\text{Enc}}_{R}^{i}⊕{\text{Enc}}_{S}^{i}$	
${\text{Dec}}_{reg}^{4}$	MEnc^5^	MEnc^4^	DeConv_2,64_, ReLU, ResConc, (Conv_3,64_, ReLU)*3, AddId	(18, 26, 18, 64)
${\text{Dec}}_{reg}^{3}$	${\text{Dec}}_{reg}^{4}$	MEnc^3^	DeConv_2,32_, ReLU, ResConc, (Conv_3,32_, ReLU)*3, AddId	(36, 52, 36, 32)
${\text{Dec}}_{reg}^{2}$	${\text{Dec}}_{reg}^{3}$	MEnc^2^	DeConv_2,16_, ReLU, ResConc, (Conv_3,16_, ReLU)*2, AddId	(72, 104, 72, 16)
${\text{Dec}}_{reg}^{1}$	${\text{Dec}}_{reg}^{2}$	MEnc^1^	DeConv_2,8_, ReLU, ResConc, (Conv_3,8_, ReLU), AddId	(144, 208, 144, 8)
${\text{Dec}}_{reg}^{0}$	${\text{Dec}}_{reg}^{1}$		Conv_1,3_, Sigmoid	(144, 208, 144, 3)

The sub-architectures are grouped into blocks, one per table line, whose names are indicated in the first column. Each block processed a forward signal as input identified by the second column. Additionally, both decoders have residual connections from different stages of the encoder, identified by the third column. The blocks are made of a set of successive operations where Conv_w, f_ (resp. DeConv_w, f_) stands for a convolutional (resp. deconvolutional) layer with weight size w × w × w and f filters, ReLU—Rectified Linear Unit, AddId—intra-block residual connection with the output of the first activated convolution of the corresponding block, ResConc—encoder to decoder residual connection from the output of the third column block to the current signal, Softmax and Sigmoid—finale output activation. * indicates successive repetition of the previous operations in parenthesis. For convolutions and deconvolutions layers, strides is 1 × 1 × 1 except for the Conv_2, ·_ which is 2 × 2 × 2. The first layer of the registration decoder indicates the merging operation of the source signal and the reference signal, which are obtained by inferring them successively in the encoder; ⊕ Indicates elementwise subtraction or channelwise concatenation of the source and reference list of tensors (forward network signal and four residual connection signals). The last column indicates each block output shape (channels last).

### 2.5. Optimization

The network is trained to minimize the segmentation and registration loss functions jointly. For the segmentation task the loss function is summarized in Equation (1). For registration, the classical optimization scheme is to minimize the Frobenius norm between the *R* and *D* image intensities:

(2) $$\begin{array}{r} L_{reg}=||\left(R-D\right)|{|}^{2}+\alpha ||\text{Ψ}-{\text{Ψ}}_{I}|{|}_{1} \end{array}$$

Here, in order to better achieve overall registration, the Frobenius norm within the regions predicted to be tumors is excluded from the loss function. We argue that by doing this, the model does not focus on tumor regions, which might produce very high norm due to their texture, but rather focuses on the overall registration task by looking at regions outside the tumor which contain information more pertinent to the alignment of the volumes. Here we should mention that on ${\hat{S}}_{seg}$ we apply the same displacement grid as on *S*, resulting in $D_{seg}=W\left({\hat{S}}_{seg},G\right)$. Further, let ${\hat{R}}_{seg}^{0}$ and $D_{seg}^{0}$ be binary volumes indicating the voxels which are predicted to be outside any segmented regions. Then, the registration loss can be written as

(3) $$\begin{array}{r} L_{reg}^{⋆}=||\left(R-D\right)\cdot D_{seg}^{0}\cdot {\hat{R}}_{seg}^{0}|{|}^{2}+\alpha ||\text{Ψ}-{\text{Ψ}}_{I}|{|}_{1} \end{array}$$

where · is the element-wise multiplication, ||·||^2^ indicates the Frobenius norm, Ψ_*I*_ is the spatial gradient of the identity deformation and α is the regularization hyperparameter. The use of regularization on the displacements Ψ is essential in order to constrain the network to predict smooth deformation grids that are anatomically more meaningful while at the same time regularize the objective function toward avoiding local minimum.

Finally the final optimization of the framework is performed by the joint optimization of the segmentation and registration loss functions

$$\begin{array}{l} L=L_{reg}+\beta L_{seg} \end{array}$$

where β is a weight that indicates the influence of each of the components on the joint optimization of the network and was defined after grid search.

For the training process, the initial learning rate was 2 · 10^−3^ and subdued by a factor of 5 if the performance on the validation set did not improve for 30 epochs. The training procedure stops when there is no improvement for 50 epochs. The regularization weights α and β were set to 10^−10^ and 1 after grid search. As training samples, random pairs among all cases were selected with a batch size limited to 2 due to the limited memory resources on the GPU. The performance of the network was evaluated every 100 batches, and both proposed models converged after nearly 200 epochs. The overall training time was calculated to ~20 h, while the time for inference of one pair, using four different modalities was ~3 s, using an NVIDIA GeForce GTX 1080 Ti GPU.

### 2.6. Datasets

**Figure 2:**
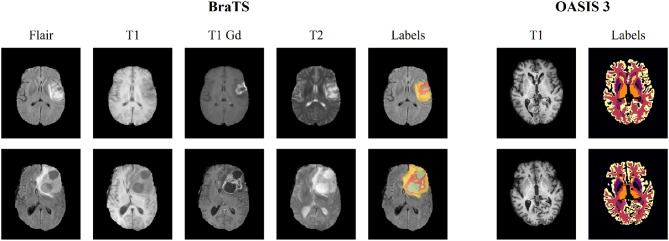
Illustration of a slice extracted from two different subjects for both BraTS 2018 and OASIS 3 datasets. The BraTS dataset consists of four modalities (T1, T1 gadolinium [T1 Gd], T2, T2 FLAIR [Flair]), along with voxelwise annotations for the three tumor tissue subclasses depicting the overall extent of tumors. OASIS 3 consists of a single T1 modality, and images are provided with voxelwise annotations for 47 different normal brain structures for patients without brain tumors.

We evaluated the performance of our method using two publicly available datasets, namely the Brain Tumor Segmentation (BraTS) (Bakas et al., 2018) and Open Access Series of Imaging Studies (OASIS 3) (Marcus et al., 2010) datasets. BraTS contains multi-institutional pre-operative MRI scans of whole brains with visible gliomas, which are intrinsically heterogeneous in their imaging phenotype (shape and appearance) and histology. The MRIs are all pre-operative and consist of four modalities, i.e., 4 3D volumes, namely (a) a native T1-weighted scan (T1), (b) a post-contrast Gadolinium T1-weighted scan (T1Gd), (c) a native T2-weighted scan (T2), and (d) a native T2 Fluid Attenuated Inversion Recovery scan (T2-FLAIR). The BraTS MRIs are provided with voxelwise ground-truth annotations for five disjoint classes denoting (a) the background, (b) the necrotic and non-enhancing tumor core (NCR/NET), (c) the GD-enhancing tumor (ET), (d) the peritumoral edema (ED) as well as invaded tissue, and finally (e) the rest of the brain, i.e., brain with no abnormality nor invaded tissue. Each center was responsible for annotating their MRIs, with a central validation by domain experts. We use the original dataset split of BraTS 2018 which contains 285 training samples and 66 for validation. In order to perform our experiments, we split this training set into three parts, i.e., train, validation and test sets (199, 26, and 60 patients, respectively), while we used the 66 unseen cases on the platform to report the performance of the proposed and the benchmarked methods. Moreover, and especially for the registration task, we evaluated the performance of the models trained on BraTS on the OASIS 3 dataset to test the generalization of the method. We extract from this dataset a subset of 150 subjects which were characterized as either non-demented or with mild cases of Alzheimer's disease (AD) using the Clinical Dementia Rating (CDR). Each scan is made of 3–4 individual T1-weighted MRIs, which has been intended to reduce the signal-to-noise ratio visible with single images. The scans are also provided with annotations for 47 different structures for left and right side of the brain generated with FreeSurfer. Some samples of both datasets can be seen in Figure 2.

The same pre-processing steps have been applied for both datasets. MRIs were resampled to voxels of volume 1 mm^3^ using trilinear interpolation. Each scan is then centered by automatically translating their barycenter to the center of the volume. Ground-truth masks of training and validation steps were accordingly translated. Each modality of each scan has been standardized, i.e., the values of the voxels of the 3D subscans were of zero mean and of unit variance. This normalization step is done independently for each patient and for each channel in order to equally consider each channel since modalities have voxels values in completely different ranges. Finally, these consequent scans are cropped into (144, 208, 144) sized volumes.

### 2.7. Statistical Evaluations

Our contributions in this study are three-fold: (i) a multi-task scheme for joint segmentation and registration; (ii) an encoding scheme followed by a fusion scheme in the latent space to aggregate information from the pair of images; and (iii) a loss formulation (Equation 3) that relaxes the registration constraints in the tumoral regions. In this section, we present our extensive experiments to demonstrate the soundness of our method.

#### 2.7.1. Comparison With Competing Methods

To demonstrate the importance of each component of our method, we performed multiple experiments to evaluate performance for both registration and segmentation tasks by removing one or more components. In particular, we evaluated 2 merging operators—subtraction and concatenation. The resulting models are henceforth referred to as “Proposed concatenation with $L_{reg}^{⋆}$” and “Proposed subtraction with $L_{reg}^{⋆}$,” respectively. We further evaluated the importance of the proposed loss formulation, reporting the performance of the models without including it in the total loss. This model is called “w/o $L_{reg}^{⋆}$.” Finally, we also evaluated the performance of the method without the segmentation decoder, which is reported as “Proposed concatenation only reg.” and “Proposed subtraction only reg.,” which again did not use $L_{reg}^{⋆}$.

We also benchmark baseline methods, without any of the proposed contributions. Since our deep learning architecture is derived from the Vnet (Milletari et al., 2016), this model is used as baseline for segmentation. This comparison seems fair since the fully proposed approach can be seen as a Vnet for the task of segmentation: the shared encoder and the proposed loss are primarily designed for registration, and have no direct impact on the segmentation apart from the features learnt in the encoder. For completeness, the top performing results on the BraTS (Bakas et al., 2018) challenge are reported, although we argue that the comparison is unfair since our deep learning architecture is entirely based on the Vnet (Milletari et al., 2016), which is not specifically designed to perform well on the BraTS segmentation task. Finally, we also report the performance of Voxelmorph (Dalca et al., 2018), a well-performing brain MRI registration neural network-based approach, although their entire deep learning structure as well as their grid formulation is different.

#### 2.7.2. Performance Assessment

For performance assessment of the segmentation task, we reported the Dice coefficient metric and Hausdorff distance to measure the performance for the tumor classes Tumor Core (TC), Enhancing Tumor (ET), and Whole Tumor (WT) as computed and provided from the BraTS submission website. These classes are the ones used in the BraTS challenge (Bakas et al., 2018), but differ from the original ones provided in the BraTS dataset: TC is the same as the one labeled in the BraTS dataset for necrotic core (NCR/NET), ET is the disjoint union of the original classes NCR/NET and ET, while WT refers to the union of all tumoral and invaded tissues.

For the registration, we evaluated the change on the tumor area together with the Dice coefficient metric for the following categories of the OASIS 3 dataset: brain stem (BS), cerebrospinal fluid (CSF), 4th ventricle (4V), amygdala (Am), caudate (Ca), cerebellum cortex (CblmC), cerebellum white matter (CblmWM), cerebral cortex (CeblC), cerebral white matter (CeblWM), hippocampus (Hi), lateral ventricle (LV), pallidum (Pa), putamen (Pu), ventral DC (VDC), and 3rd ventricle (3V) categories. Here we should mention that for the experiments with the OASIS 3 dataset, we performed a training only with the T1-weighted MRIs of the BraTS dataset, in order to match the available modalities of the OASIS 3 dataset. This evaluation is important as (i) BraTS does not provide anatomical annotations in order to evaluate quantitatively the registration performance and (ii) the generalization of the proposed method on an unseen dataset is evaluated. For the registration of tumor tissues, which might not exist in the source or reference MRIs, we expect the model to register tumor areas while maintaining their geometric properties. In particular, we do not really expect the tumor areas to stay completely unchanged. However, we expect that the volume of the different tumor types would change with a ratio similar to the one that the entire source to the reference volume changes. We calculate this ratio by computing $\frac{D_{seg}^{j}}{S_{seg}^{j}}$ where *j* = {0, 1, 2, 3} corresponds to the entire brain and the different tumor classes (NCR/NET, ET, and ED). We then assess the change of the tumor by calculating the absolute value of the difference between *j* = 1 and every other tumor class. Ideally, we expect a model which preserves the tumor geometry and shape during inference to present a zero difference between the entire brain and tumor class ratio. We independently calculate this difference for each tumor class in order to monitor the behavior of each class, but also after merging the entire tumor area.

For statistical significance evaluations between any two methods, we compute independent *t*-tests as presented in Rouder et al. (2009), defining as null hypothesis the evaluation metrics of the two populations to be equal. We then report the associated *p*-value, and the Cohen's d (Rice and Harris, 2005), which we use to measure the effect size. Such statistical significance evaluation is reported in the form (*t*(*n*); *p*; *d*) where *n* is the number of samples for each population, *t*(*n*) is the *t*-value, *p* is the *p*-value and *d* is Cohen's d. We defined the difference of two population means is statistically significant if the associated *p*-value is lower than 0.005, and consider, as a rule of thumb, that a value of *d* of 0.20 indicates small effect size, 0.50 for medium effect size and 0.80 for large effect size. All of the results in this paper have been computed on unseen testing sets, and the performance of all benchmarked models has been assessed once.

For rigor and for each *t*-test conducted, we ensure the following assumptions are met by the underlying distributions: observations are independent and identically distributed, the outcome variable follows a normal distribution in the population (with Jarque and Bera, 1980), and the outcome variable has equal standard deviations in two considered (sub)populations [using Levene's test (Schultz, 1985)]. Finally, when comparing two populations, each made of several subpopulations, we merge such subpopulations into a single set, then compute *t*-tests on the obtained two gathered-populations.

## 3. Results

### 3.1. Evaluation of the Segmentation

Segmentation results for the tumor regions are displayed in Table 2 for the case of the same autoencoder architecture trained only with a segmentation decoder (Baseline *segmentation*) and the proposed method using different merging operations and with or without $L_{reg}^{⋆}$. One can observe that all evaluated methods perform quite similarly with Dice higher than 0.66 for all the classes and models. The *baseline segmentation* model reports slightly better average Dice coefficient and average Haussdorf distance measurements, with an average Dice 0.03 higher, and an average Hausdorff95 distance 0.6 higher than the proposed with concatenation merging operator, although none of these differences are found statistically significant as indicated in Table 3. In particular, for Dice, the minimum received *p*-value was *p* = 0.24, reported between *baseline segmentation* and *proposed concatenation with* $L_{reg}^{⋆}$ together with an associated Cohen's *d* = 0.21 indicating a small size effect. Similarly, for Hausdorff95, the minimum received *p*-value was *p* = 0.46, reported this time between *baseline segmentation* and *proposed concatenation w/o* $L_{reg}^{⋆}$ with *d* = 0.13 also indicating a small size effect. These numbers show that the means differences between those two models and any other two models are not statistically significant. This is very promising if we take into account that our proposed model is learning a far more complex architecture addressing both registration and segmentation, with the same volume of training data without significant drop of the segmentation performance.

**Table 2:** Quantitative results of the different methods on the segmentation task on the BraTS 2018 validation dataset.

	**Average**		**Dice**			**Hausdorff95**		
**Method**	**Dice**	**Hausdorff95**	**ET**	**WT**	**TC**	**ET**	**WT**	**TC**
Baseline segmentation	0.79 ± 0.29	7.0 ± 9.6	0.73 ± 0.29	0.87 ± 0.13	0.75 ± 0.24	4.7 ± 8.2	7.2 ± 9.4	9.2 ± 8.9
Proposed								
Concatenation w/o $L_{reg}^{⋆}$	0.74 ± 0.29	8.3 ± 10.4	0.70 ± 0.29	0.87 ± 0.11	0.65 ± 0.29	6.2 ± 9.8	7.8 ± 11.1	11.3 ± 7.1
Concatenation with $L_{reg}^{⋆}$	0.73 ± 0.29	7.6 ± 9.9	0.68 ± 0.30	0.87 ± 0.12	0.66 ± 0.28	6.3 ± 9.9	5.6 ± 4.2	10.8 ± 6.6
Subtraction w/o $L_{reg}^{⋆}$	0.76 ± 0.27	7.8 ± 10.3	0.71 ± 0.28	0.88 ± 0.10	0.70 ± 0.24	6.5 ± 10.8	7.4 ± 11.0	10.0 ± 7.4
Subtraction with $L_{reg}^{⋆}$	0.76 ± 0.27	7.9 ± 10.1	0.71 ± 0.29	0.88 ± 0.10	0.69 ± 0.25	5.8 ± 9.6	7.7 ± 11.5	11.1 ± 8.3

*Dice and Hausdorff95 are reported for the three classes Whole Tumor (WT), Enhancing Tumor (ET), and Tumor Core (TC) together with their average values. Results are reported with mean across patients (MRIs) along with the associated standard deviation. We upload our predictions on the official leaderboard of the validation set (66 patients)*.

**Table 3:** Statistical significance of the proposed methods with Milletari et al. (2016) on the BraTS segmentation task.

	**Average**		**Dice**			**Hausdorff95**		
**Method**	**Dice**	**Hausdorff95**	**ET**	**WT**	**TC**	**ET**	**WT**	**TC**
Baseline segmentation	1.00	1.00	1.00	1.00	1.00	1.00	1.00	1.00
Proposed								
Concatenation w/o $L_{reg}^{⋆}$	0.32	0.46	0.55	1.00	0.03	0.34	0.74	0.14
Concatenation with $L_{reg}^{⋆}$	0.24	0.72	0.33	1.00	0.05	0.31	0.21	0.24
Subtraction w/o $L_{reg}^{⋆}$	0.55	0.65	0.69	0.62	0.24	0.28	0.91	0.58
Subtraction with $L_{reg}^{⋆}$	0.55	0.60	0.69	0.62	0.16	0.48	0.79	0.21

*For each model (line) and each performance measure (column), the displayed value is the p-value, up to two significant figures, of the statistical significance between the model and Milletari et al. (2016) for the corresponding measure (Dice or Hausdorff95) on the corresponding tumor class (ET, WT, TC, or the union of the three latter in the two columns Average) on the 66 testing samples of BraTS. No p-values are statistically significant between all of the proposed variants and Milletari et al. (2016). Blue line represents the reference model, red cells indicate no statistical significant p-values (cutoff 0.005)*.

The superiority of the *baseline segmentation* seems to be presented mainly due to higher performance for the TC class [*baseline segmentation* and *proposed subtraction with* $L_{reg}^{⋆}$: *t*_(66)_ = 1.41; *p* = 0.16; *d* = 0.24]. Moreover, the concatenation operation seems to perform slightly better for the tumor segmentation than the subtraction, with at least 0.02 improvement for average Dice coefficient, although this improvement is not statistically significant [*proposed concatenation with* $L_{reg}^{⋆}$ and *proposed subtraction with* $L_{reg}^{⋆}$: *t*_(66)_ = 0.62; *p* = 0.53; *d* = 0.11].

Moreover, even if one of the main goals of our paper is the proper registration of the tumoral regions, we perform a comparison with the two best performing methods presented in BraTS 2018 (Isensee et al., 2018; Myronenko, 2018) evaluated on the validation dataset of BraTS 2018. In particular, the Myronenko (2018) reports an average dice of 0.82, 0.91, and 0.87 for ET, WT, and TC, respectively, while Isensee et al. (2018) reports 0.81, 0.91, and 0.87. Both methods outperform our proposed approach on the validation set of BraTS 2018 by integrating novelties specifically designed to the tumor segmentation task of BraTS 2018. In this study, we based our architecture in a relatively simple and widely used 3D fully convolutional network (Milletari et al., 2016) although different architectures with tumor specific components (trained on the evaluated tumor classes), trained on more data (similar to the ones that are used from Isensee et al., 2018), or even integrating post-processing steps can be easily integrated boosting considerably the performance of our method.

**Figure 3:**
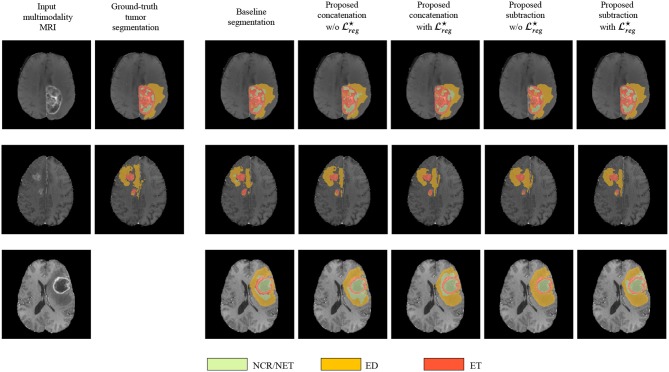
The segmentation maps produced by the different evaluated methods displayed on post-contrast Gadolinium T1-weighted modalities. We present the provided segmentation maps both on our test dataset and on the BraTS 2018 validation dataset. NCR/NET, necrotic core; ET, GD-enhancing tumor; ED, peritumoral edema.

Finally, in Figure 3 we represent the ground truth and predicted tumor segmentation maps comparing the *baseline segmentation* and our proposed method using the different components and merging operators. We present three different cases, two from our custom test set, on which we have the ground truth information and one from the validation set of the BraTS submission page. One can observe that all the methods provide quite accurate segmentation maps for all the three tumor classes.

### 3.2. Evaluation of the Registration

#### 3.2.1. Evaluation on Anatomical Structures

The performance of the registration has been evaluated on an unseen dataset with anatomical information, namely OASIS 3. In Table 4 the mean and standard deviation of the Dice coefficient for the different evaluated methods are presented. With rigid we indicate the Dice coefficient after the translation of the volumes such that the center of the brain mass is placed in the center of the volume. It can be observed that the performance of the evaluated methods are quite similar something which indicates that the additional tumor segmentation decoder does not decrease the performance of the registration. On the other hand, it provides additional information about the areas of tumor in the image. From our experiments, we show that the proposed formulation can provide registration accuracy similar to the recent state-of-the-art deep learning based methods (Dalca et al., 2018) with approximately the same average Dice values, that is 0.50 for (Dalca et al., 2018) and 0.49 for all but one of the proposed variants. Moreover, again this difference in the performance between (Dalca et al., 2018) and the proposed method is not statistically significant with *t*_(150)_ = 0.64; *p* = 0.52; *d* = 0.07. From our comparisons, the only significant difference on the evaluation of the registration task was reported between the proposed method *concatenation only reg*. with an average difference of dice reaching 0.05% and with maximum *p*-values calculated with *Proposed concatenation with* $L_{reg}^{⋆}$ [*t*_(200)_ = 3, 33; *p* < 10^−3^; *d* = 0, 38]. From our experiments, we saw that the merging operation affects the performance of the *only reg*. model a lot, with the concatenation reporting the worst average dice of all the methods.

**Table 4:** The mean and standard deviation of the dice coefficient for the 15 different classes of OASIS 3 dataset for the different evaluated methods.

**Method**	**BS**	**CSF**	**CblmC**	**CblmWM**	**CeblWM**	**Pu**	**VDC**	**Pa**	**Ca**	**LV**	**Hi**	**3V**	**4V**	**Am**	**CeblC**	**Average**
Rigid	0.58 ± 0.15	0.39 ± 0.11	0.46 ± 0.13	0.40 ± 0.14	0.49 ± 0.05	0.44 ± 0.13	0.47 ± 0.13	0.35 ± 0.17	0.27 ± 0.15	0.40 ± 0.13	0.34 ± 0.13	0.39 ± 0.17	0.15 ± 0.15	0.24 ± 0.18	0.36 ± 0.04	0.38 ± 0.13
Voxelmorph	0.69 ± 0.12	**0.46** ± **0.13**	**0.63** ± **0.11**	**0.57** ± **0.13**	**0.73** ± **0.083**	0.42 ± 0.14	0.5 ± 0.11	0.33 ± 0.14	0.42 ± 0.17	0.62 ± 0.14	0.38 ± 0.13	**0.53** ± **0.18**	**0.32** ± **0.23**	0.25 ± 0.17	**0.6** ± **0.084**	**0.5** ± **0.14**
Proposed																
Concatenation																
Only reg.	0.65 ± 0.15	0.34 ± 0.1	0.58 ± 0.11	0.48 ± 0.14	0.6 ± 0.056	0.46 ± 0.12	0.47 ± 0.12	0.38 ± 0.14	0.35 ± 0.15	0.54 ± 0.14	0.35 ± 0.13	0.4 ± 0.16	0.21 ± 0.17	0.27 ± 0.18	0.46 ± 0.051	0.44 ± 0.13
w/o $L_{reg}^{⋆}$	0.72 ± 0.13	0.42 ± 0.1	0.61 ± 0.11	0.51 ± 0.12	0.63 ± 0.056	0.47 ± 0.14	0.51 ± 0.12	0.37 ± 0.16	**0.44** ± **0.15**	**0.65** ± **0.13**	**0.42** ± **0.14**	0.46 ± 0.17	0.31 ± 0.22	0.31 ± 0.19	0.48 ± 0.052	0.49 ± 0.13
With $L_{reg}^{⋆}$	0.7 ± 0.15	0.44 ± 0.12	0.6 ± 0.13	0.52 ± 0.14	0.66 ± 0.06	0.47 ± 0.14	0.52 ± 0.13	0.38 ± 0.16	0.42 ± 0.16	0.65 ± 0.14	0.4 ± 0.15	0.51 ± 0.19	0.3 ± 0.22	0.28 ± 0.2	0.49 ± 0.058	0.49 ± 0.14
Subtraction																
Only reg.	0.71 ± 0.13	0.41 ± 0.1	0.61 ± 0.12	0.53 ± 0.13	0.66 ± 0.058	0.47 ± 0.12	0.5 ± 0.11	0.37 ± 0.15	0.43 ± 0.14	0.63 ± 0.12	0.4 ± 0.13	0.47 ± 0.16	0.34 ± 0.22	0.29 ± 0.19	0.49 ± 0.054	0.49 ± 0.13
w/o $L_{reg}^{⋆}$	0.7 ± 0.13	0.41 ± 0.1	0.6 ± 0.11	0.52 ± 0.12	0.65 ± 0.057	**0.48** ± **0.13**	**0.53** ± **0.11**	**0.39** ± **0.15**	0.43 ± 0.14	0.64 ± 0.13	0.41 ± 0.13	0.49 ± 0.17	0.3 ± 0.22	0.29 ± 0.18	0.48 ± 0.053	0.49 ± 0.13
With $L_{reg}^{⋆}$	**0.72** ± **0.12**	0.4 ± 0.11	0.61 ± 0.11	0.53 ± 0.12	0.64 ± 0.058	0.47 ± 0.12	0.51 ± 0.11	0.38 ± 0.15	0.41 ± 0.15	0.63 ± 0.13	0.43 ± 0.13	0.44 ± 0.17	0.3 ± 0.22	**0.33** ± **0.18**	0.48 ± 0.054	0.49 ± 0.13

*The first two rows are baseline methods. The rest of the rows present the results of our proposed method evaluating the different variants and merging operators. The names of the columns represent various brain structures, namely: brain stem (BS), cerebrospinal fluid (CSF), 4th ventricle (4V), amygdala (Am), caudate (Ca), cerebellum cortex (CblmC), cerebellum white matter (CblmWM), cerebral cortex (CeblC), cerebral white matter (CeblWM), hippocampus (Hi), lateral ventricle (LV), pallidum (Pa), putamen (Pu), ventral DC (VDC), and 3rd ventricle (3V). Bold indicates best performance per column*.

**Figure 4:**
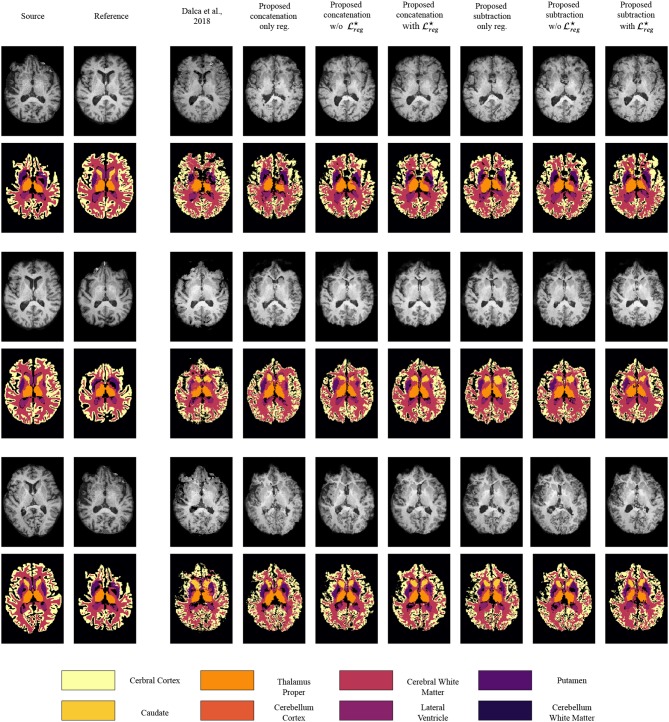
Qualitative evaluation of the registration performance for the different evaluated methods, displayed on T1 modalities. For an easier visualization, we group left and right categories and only display the following nine classes: caudate (Ca), cerebellum cortex (CblmC), cerebellum white matter (CblmWM), cerebral cortex (CeblC), cerebral white matter (CeblWM), lateral ventricle (LV), pallidum (Pa), putamen (Pu), ventral DC (VDC).

In Figure 4 we present some qualitative evaluation of the registration component, by plotting three different pairs and their registration from all the evaluated models. The first two columns of the figure depict the source and reference volumes together with their tissue annotations. The rest of the columns present the deformed source volume together with the deformed tissue annotations for each of the evaluates methods. Visually, all methods perform well on the overall shape of the brain with the higher errors in the deformed annotations being presented at the cerebral write matter and cerebral cortex classes.

Finally, we should also mention that the subjects of the OASIS 3 dataset do not contain regions with tumors. However, our proposed formulation provides tumor masks so that we could evaluate the robustness of the segmentation part. Indeed, our model for all the different combinations of merging operations and loss functions, reported a precision score of more than 0.999, indicating its robustness for the tumor segmentation task.

#### 3.2.2. Evaluation on the Tumor Areas

Even if the proposed method reports very similar performance with models that perform only registration, we argue that it addresses better the registration of the tumor areas, maintaining their geometric properties, as can be inferred in Table 5. This statement is also supported by the statistical tests we performed to evaluate the difference in performance between the methods, while registering tumor areas (Table 6). In particular, for each of the tumor classes NCR/NET, ET, and ED the difference between (Dalca et al., 2018) and the proposed method *subtraction with* $L_{reg}^{⋆}$ was significant with NCR/NET: *t*_(200)_ = 10.69; *p* < 10^−3^; *d* = 1.07—ET: *t*_(200)_ = 10.51; *p* < 10^−3^; *d* = 1.05—ED: *t*_(200)_ = 8.05; *p* < 10^−3^; *d* = 0.81. The similar behavior was obtained when the evaluation was performed by merging all 3 tumor classes into one (denoted *Combined*). Again, we reported significant differences between (Dalca et al., 2018) and the proposed method: *t*_(200)_ = 11.38; *p* < 10^−3^; *d* = 1.14.

**Table 5:** The table presents the average distance between (i) the ratio of the area of the deformed tumor mask to the area of the original tumor mask, and (ii) the ratio of area of the reference brain volume to the area of the source brain volume.

**Method**	**NCR/NET**	**ET**	**ED**	**Combined**
Dalca et al. (2018)	2.27 ± 2.68	0.67 ± 0.55	1.96 ± 3.03	0.62 ± 0.51
Proposed				
Concatenation only reg.	0.51 ± 0.61	0.26 ± 0.19	0.71 ± 0.94	0.22 ± 0.15
Concatenation w/o $L_{reg}^{⋆}$	1.35 ± 1.14	0.64 ± 0.41	1.80 ± 1.82	0.64 ± 0.42
Concatenation with $L_{reg}^{⋆}$	0.26 ± 0.20	0.26 ± 0.13	0.30 ± 0.28	0.21 ± 0.12
Subtraction only reg.	1.34 ± 0.77	0.77 ± 0.59	2.02 ± 1.65	0.68 ± 0.52
Subtraction w/o $L_{reg}^{⋆}$	1.74 ± 1.35	0.72 ± 0.72	2.38 ± 1.74	0.74 ± 0.76
Subtraction with $L_{reg}^{⋆}$	**0.24** ± **0.17**	**0.25** ± **0.13**	**0.23** ± **0.22**	**0.20** ± **0.11**

Lower values are better. The average has been calculated over 200 testing pairs from the BraTS 2018 dataset (NCR/NET, ET and ED). On top of the evaluation per tumor class, we also conduct an evaluation by merging all the tumor classes into just one class (called combined). Bold indicates best performance per column.

**Table 6:** Summary of the statistical difference between the Dalca et al. (2018) and the proposed method on the BraTS 2018 dataset for the tumor preservation task.

**Method**	**NCR/NET**	**ET**	**ED**	**Combined**
Dalca et al. (2018)	<10^−3^	<10^−3^	<10^−3^	<10^−3^
Proposed				
Concatenation only reg.	<10^−3^	0.540	<10^−3^	0.130
Concatenation w/o $L_{reg}^{⋆}$	<10^−3^	<10^−3^	<10^−3^	<10^−3^
Concatenation with $L_{reg}^{⋆}$	0.282	0.442	0.006	0.386
Subtraction only reg.	<10^−3^	<10^−3^	<10^−3^	<10^−3^
Subtraction w/o $L_{reg}^{⋆}$	<10^−3^	<10^−3^	<10^−3^	<10^−3^
Subtraction with $L_{reg}^{⋆}$	1.000	1.000	1.000	1.000

For each model (line) and each performance measure (column), the displayed value is the *p*-value (up to 3 significant figures) of the statistical significance between the model and subtraction with $L_{reg}^{⋆}$ for the tumor preservation measure on the corresponding tumor class (NCR/NET, ET, ED, and their union in the column Combined). Blue line represents the reference model, red cells indicate no statistical significant *p*-values while green cells represents statistical significant *p*-values.

To evaluate the performance of the different variants of our proposed method, we compared the performance of the proposed *subtraction with* $L_{reg}^{⋆}$ and *concatenation with* $L_{reg}^{⋆}$ that reported the best performances. Indeed, we did not find significant changes between the two different components except the edema class [*t*_(200)_ = 2.78; *p* < 10^−3^; *d* = 0.28]. Moreover, the proposed *concatenation only reg*. reports also competitive results without using the segmentation masks. In particular, even if the specific method does not report very good performance on the registration evaluated on anatomical structures (section 3.2.1), it reports very competitive performance on the *Combined* and the smallest in size tumor class (*ET*). However, for the other two classes the difference on the performance that it reports in comparison to the proposed variant *subtraction with* $L_{reg}^{⋆}$ is significant different: NCR/NET: *t*_(200)_ = 6, 03; *p* < 10^−3^; *d* = 0, 60—ED: *t*_(200)_ = 7, 03; *p* < 10^−3^; *d* = 0, 70. Here we should mention that even though *subtraction only reg*. works very well for the registration of the anatomical regions (section 3.2.1), it reports one of the worst results about tumor preservation, with values close to the ones reported by Dalca et al. (2018). This indicates again that the *only reg*. model is highly sensitive to the merging operation and it cannot simultaneously provide good performance on tumor areas and registration of the entire volume, proving its inferiority to the proposed method using the *with* $L_{reg}^{⋆}$.

Independently of the merging operation with both registration and segmentation tasks, i.e., with or without $L_{reg}^{⋆}$, we find that the proposed approach works significantly better in preserving tumor areas when optimized with $L_{reg}^{⋆}$ than without [NCR/NET: *t*_(200)_ = −14.33; *p* < 0.005; *d* = 1.43—ET: *t*_(200)_ = −9.99; *p* < 0.005; *d* = 1.00—ED: *t*_(200)_ = −14.17; *p* < 0.005; *d* = 1.42—Combined: *t*_(200)_ = −10.94; *p* < 0.005; *d* = 1.09].

**Figure 5:**
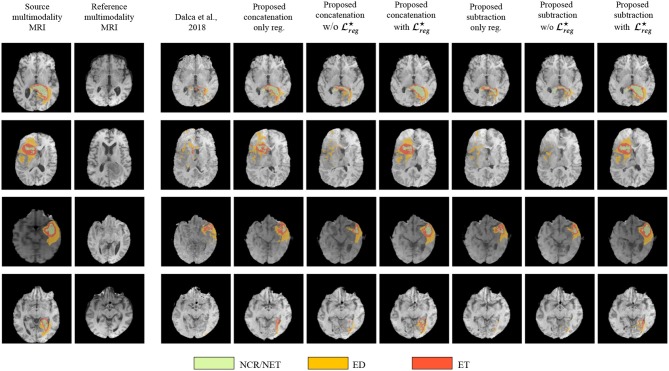
Qualitative evaluation of the tumor deformation of the different evaluated methods, displayed on T1 modalities. Each line is a sample, with source MRI in the first column to be registered on reference MRI in the second column. BraTS ground-truth annotations are plotted onto the source MRI. Seven models are benchmarked, one for each of the remaining columns which display the result of applying the predicted grid onto the source MRI. For each model and each line, the source ground-truth annotation masks of the source MRI were also registered with the predicted deformation grid, and the consequently obtained deformed ground-truth were plotted onto each deformed source MRI to illustrate the impact of all methods regarding the preservation of tumor extent.

Figure 5 presents some qualitative examples from the BraTS 2018 to evaluate the performance of the different methods. The first two columns present the pair of images to be registered and segmented and the rest of the columns the deformed source image with the segmented tumor region superimposed. One can observe that the most of the methods that are based only on registration (Dalca et al., 2018, proposed concatenation and subtraction *only reg*.) together with the proposed concatenation and subtraction *w/o* $L_{reg}^{⋆}$ do not preserve the geometry of the tumor, tending to significantly reduce the area of tumor after registration, or intermix the different types of tumor. On the other hand the behavior of the proposed *with* $L_{reg}^{⋆}$ seems to be much better, with the tumor area properly maintained in the deformed volume.

**Figure 6:**
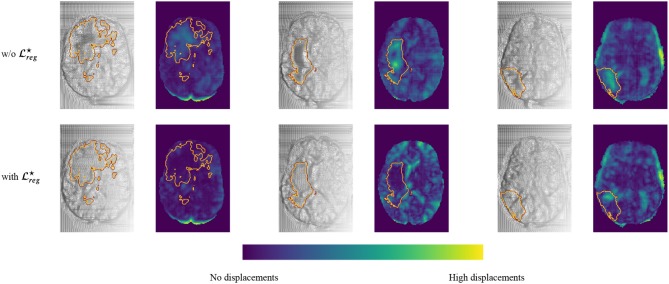
Comparison of the registration grid of the proposed model using the subtraction operation with and w/o $L_{reg}^{⋆}$. This figure is obtained by sampling three random pairs of test patients, and computing the predicted registration fields, which are displayed by line for the two models, and in consecutive columns, one for each of the three dimensions, showing the registration field as a warped grid (grayscale) and as a colored map obtained by computing its norm pixelwise (blue-green map). Furthermore, the contour of the Whole Tumor is plotted on top of each image, obtained from the ground truth segmentation.

Moreover, in Figure 6 we provide a better visualization for the displacement grid inside the tumor area, highlighting the importance of Equation (3). Indeed, one can observe that the displacements inside the tumor area are much smoother and relaxed when we use the information about the tumor segmentation.

## 4. Discussion

In this study, we proposed a novel deep learning based framework to address simultaneously segmentation and registration. The framework combines and generates features, integrating valuable information from both tasks within a bidirectional manner, while it takes advantage of all the available modalities, making it quite robust and generic. The performance of our model indicates highly promising results that are comparable to recent state-of-the-art models that address each of the tasks separately (Dalca et al., 2018). However, we reported a better behavior of the model in the proximity of tumor regions. This behavior has been achieved by training a shared encoder that generates features that are meaningful for both registration and segmentation problems. At the same time, these two problems have been coupled in a joint loss function, enforcing the network to focus on regions that exist in both volumes.

Even if we could not do a proper comparison with Parisot et al. (2012) which shares similar concepts, our method provides very good improvements. In particular, we train both problems at the same time, without using pre-calculated classification probabilities. The method proposed in Parisot et al. (2012) is based on a pre-calculated classifier indicating the tumoral regions. The authors provided their segmentation results by adapting Gentle Adaboost algorithm and using different features including intensity values, texture, such as Gabor filters and symmetry. After training the classifier they defined an MRF model to optimize their predictions by taking into account pairwise relations. By adopting this strategy, the used probabilities for the tumoral regions are not optimized simultaneously with the registration, something that it is not the case in our methodology. In particular, by sharing representation between the registration and segmentation tasks we argue that we can create features that are more complex and useful sharing information that comes from both problems. By using a deep learning architecture that is end-to-end trainable, we are able to extract features that are suitable to deal with both problems automatically. Moreover, our implementation is modular and scalable permitting easy integration of multiple modalities, something that is not so straightforward with Parisot et al. (2012) as it is more complicated to adapt and calculate the different similarity measures and classifiers taking into account all these modalities. Finally, we should mention that our method takes advantage of GPU implementation needing only a few seconds in order to provide segmentation and displacement maps while the method in Parisot et al. (2012) needs ~6 min.

Both qualitative and quantitative evaluations of the proposed architecture highlight its great potentials reporting more than 0.66 Dice coefficient for the segmentation of the different tumor areas, evaluated on the publicly available BraTS 2018 validation set. Our joint formulation reported performance similar to the model trained only for segmentation, while simultaneously addressing the registration problem. Moreover, both concatenation and subtraction operators report similar performances, an expected result for the specific segmentation task, since the merging operation is mainly used on the registration decoder, even if it affects the learned parameters of the encoder and thus indirectly the segmentation decoder.

Concerning the comparison between top performing tumor segmentation methods, although our formulation underperforms the winning methods of BraTS 2018, we want to highlight two major points. First of all, our formulation is modular in the sense that different network architectures with optimized components for tumor segmentation can be evaluated depending on the application and the goals of the problem. For our experiments we chose a simple VNet architecture (Milletari et al., 2016) proving that the registration components do not significantly hinder the segmentation performance and indicating the soundness of our method however any other encoder decoder architecture can be used and evaluated. Secondly, the main goal of our method was the proper registration and segmentation of the tumoral regions together with the rest of the anatomical structures and that was the main reason we did not optimize our network architecture according to the winning methods of BraTS 2018. However, we demonstrated that with a very simple architecture, we can register properly tumoral and anatomical structures while segmenting with more than 76% of Dice the tumoral regions.

Continuing with the evaluation of the registration performance, once more the joint multi task framework reports similar and without statistical difference performance with formulations that address only the registration task evaluated on anatomical regions that exist on both volumes. However, we argue that abnormal regions registration is better addressed both in terms of qualitative and quantitative metrics. Moreover, from our experiments we observed that subtraction of the coding features of the tumors reports higher performances for the registration of the tumor areas. This indicates that the subtraction can capture and code more informative features for the registration task. What is more, we achieved very good generalization for all the deep learning based registration methods, as they reported very stable performance in a completely unseen dataset (part of the OASIS3).

Even if, from our experiments, the competence of our proposed method for both registration and segmentation tasks is indicated, we report a much better performance for the registration of the tumoral regions. In particular, in one joint framework we were able to produce efficiently and accurately tumor segmentation maps for both source and reference images together with their displacement maps that register the source volume to the reference volume space. Our experiments indicated that the proposed method with the $L_{reg}^{⋆}$ variant register properly the anatomical together with the tumoral regions with statistical significance compare to the rest of the methods for the latter. Both qualitative and quantitative evaluations of the different components indicate the superiority of the with $L_{reg}^{⋆}$ variant of the proposed method for brain MRI registration with tumor extent preservation. Using such a formulation, the network focus on improving local displacements on tissues anywhere in the common brain space instead of minimizing the loss within the tumoral regions, which are empirically the regions with the highest registration errors. Consequently, the network improves its registration performance on non-tumor regions (as discussed in section 3.2.1), while also relaxing the obtained displacements inside those predicted tumor regions.

Some limitations of our method include the number of parameters that have to be tuned during the training due to the multi task nature of our formulation, namely α and β that affect the performance of the network. Moreover, due to the multimodal nature of the input and the two decoders, the network cannot be very deep due to GPU memory limitations.

Although the pipeline was built using different patients for the registration task as a proof of concept, such tool could have numerous applications in clinical practice, especially when applied in different images acquired from the same patient. Regarding the radiotherapy treatment planning, several studies have shown that significant changes of the targeted volumes in the brain occurred during radiotherapy raising the question of replanning treatment to reduce the amount of healthy brain irradiated in case of tumor reduction, or to re-adapt the treatment for brain tumors that grow during radiation (Champ et al., 2012; Yang et al., 2016; Mehta et al., 2018). Since MR-guided linear accelerator will offer the opportunity to acquire daily images during RT treatment, the proposed tool could help with automatic segmentation and image registration for replanning purposes, and it could also allow accurate evaluation of the dose delivered in targeted volumes and healthy tissues by taking into account the different volume changes. Moreover, while changes of imaging features under treatment is known to be associated with treatment outcomes in several cancer diseases (Vera et al., 2014; Fave et al., 2017), the registration grid computed from two same-patient acquisitions realized at different times allows an objective and precise evaluation of the tumor changes.

Future work involves a better modeling of the prior knowledge through a more appropriate geometric modeling of tumor proximity that encodes more accurately the registration errors in these areas. This modeling can be integrated into the existing formulation with some additions specific to tumor losses that will further constrain its change. Moreover, we have noticed that the use of Fobenius norm during the training of the registration part is very sensitive to artifacts in the volume, preventing the network process from being completely robust. In the future, we aim to evaluate the performance of the proposed framework using adversarial losses in order to better address multimodal cases. Finally, means to automatically obtain the training parameters α and β would be investigated.

## Data Availability Statement

Publicly available datasets were analyzed in this study. This data can be found here: https://www.med.upenn.edu/sbia/brats2018/data.html, https://www.oasis-brains.org/.

## Author Contributions

TE, ML, MV, NP, and ED designed the research. TE, ML, and MV performed the research, analyzed and interpreted the data, and wrote the paper. TE, ML, MV, EA, EB, AC, SCha, SChr, MS, RS, CR, HT, NP, and ED revised and approved the paper.

### Conflict of Interest

The authors declare that the research was conducted in the absence of any commercial or financial relationships that could be construed as a potential conflict of interest.
